# Opsoclonus-Myoclonus-Ataxia Syndrome Related to Pembrolizumab Treatment for Invasive Ductal Carcinoma: A Case Report

**DOI:** 10.7759/cureus.104726

**Published:** 2026-03-05

**Authors:** Ananta Sriram, Laura Polhemus, Wilson Rodriguez, Divya Singh, Jafar Kafaie

**Affiliations:** 1 Medicine, Saint Louis University School of Medicine, St. Louis, USA; 2 Neurology, Saint Louis University Hospital, St. Louis, USA; 3 Neurology, Saint Louis University School of Medicine, St. Louis, USA

**Keywords:** immune checkpoint inhibitor, neurology case report, oncology, opsoclonus-myoclonus-ataxia syndrome, pembrolizumab side effect, triple-negative breast cancer

## Abstract

Immune checkpoint inhibitors (ICIs) such as pembrolizumab have become integral to the treatment of various metastatic malignancies. However, their use is associated with a growing spectrum of immune-related adverse events, including rare neurological complications. This report adds to the limited existing literature on ICI-associated opsoclonus-myoclonus-ataxia syndrome (OMAS) and highlights the importance of prompt recognition and multidisciplinary management of such cases.

We present a case of a 67-year-old female who presented with rapid, chaotic eye movements, truncal ataxia, and full-body myoclonus two months after beginning a combined immunotherapy and chemotherapy regimen of paclitaxel, carboplatin, and pembrolizumab for triple-negative invasive ductal carcinoma. The case highlights pembrolizumab, an ICI targeting the programmed death-1 receptor, as a cause of adult-onset OMAS. In our discussion, we expand upon the mechanism of action of ICIs, notably the immune pathways underlying their use. We follow this with how this immunotherapy causes damage to peripheral tissues, focusing on the neurological side effects. We finish by discussing currently implemented treatment options for patients who experience these adverse events, and future directions in the field of autoimmune and paraneoplastic neurology.

This case describes the onset of OMAS following pembrolizumab therapy in a patient with triple-negative breast cancer, a rare but clinically significant adverse event. As ICIs are increasingly used, clinicians must remain vigilant for uncommon neurological immune-related adverse events. Early recognition and immunosuppressive treatment can mitigate long-term sequelae. This case underscores the need for further research and interdisciplinary awareness in the evolving field of autoimmune and paraneoplastic neurology.

## Introduction

Opsoclonus-myoclonus-ataxia syndrome (OMAS) is a rare neurological disorder characterized by chaotic eye movements, action-induced or spontaneous myoclonus, and cerebellar ataxia. In adults, OMAS is classically associated with paraneoplastic syndromes, most commonly small-cell lung cancer or breast cancer, or less frequently with autoimmune or post-infectious etiologies [1,2]. As immune checkpoint inhibitors (ICIs) have become increasingly incorporated into cancer treatment regimens, reports of immune-related adverse events (IRAEs) affecting the nervous system have expanded [2]. However, OMAS, as a neurological complication of ICIs, remains exceedingly uncommon, with only a small number of cases described in the literature.

Pembrolizumab, a programmed death-1 (PD-1) inhibitor, has demonstrated significant therapeutic benefits in triple-negative breast cancer but carries the risk of inducing immune dysregulation [2]. This case is notable for the development of OMAS shortly after pembrolizumab initiation, in a clinical timeframe consistent with other ICI-related neurological events.

OMAS has been described as a classical paraneoplastic syndrome in breast cancer; however, ICIs may also trigger autoimmune neurological syndromes or unmask underlying paraneoplastic processes [1]. Differentiating paraneoplastic OMAS from ICI-related neurotoxicity can be challenging, particularly in patients with active malignancy receiving immunotherapy [1]. Paraneoplastic OMAS may occur independent of treatment and can be seronegative despite classic clinical features. In contrast, ICI-related neurological IRAEs typically arise within weeks to months of therapy initiation and may respond to immunosuppressive treatment following drug discontinuation [1]. This case highlights the diagnostic challenge of distinguishing paraneoplastic OMAS from ICI-related neurotoxicity, and the possibility that pembrolizumab contributed to the onset of symptoms in this patient.

We present this case to highlight an exceptionally rare manifestation of pembrolizumab-associated neurotoxicity. This report contributes to the growing understanding of ICI-related neurological syndromes, underscores the importance of early recognition and multidisciplinary management, and adds to the limited literature regarding OMAS triggered by modern cancer immunotherapies.

This article was previously presented as a meeting abstract at the 2024 American Academy of Neurology Annual Meeting on April 14, 2024.

## Case presentation

A 67-year-old White female with a history of major depressive disorder was diagnosed with invasive ductal carcinoma, which was grade 2, estrogen (ER) negative, progesterone (PR) negative, and HER2 negative. One month after diagnosis, she was initiated on a combined chemotherapy and immunotherapy regimen of paclitaxel, carboplatin, and pembrolizumab. Seven weeks after initiation (including three doses of pembrolizumab on weeks one, four, and seven), she began experiencing severe nausea, vomiting, tachycardia, lightheadedness, and paresthesia. These symptoms progressed over the next four days, prompting hospitalization for severe dehydration.

**Figure 1:**
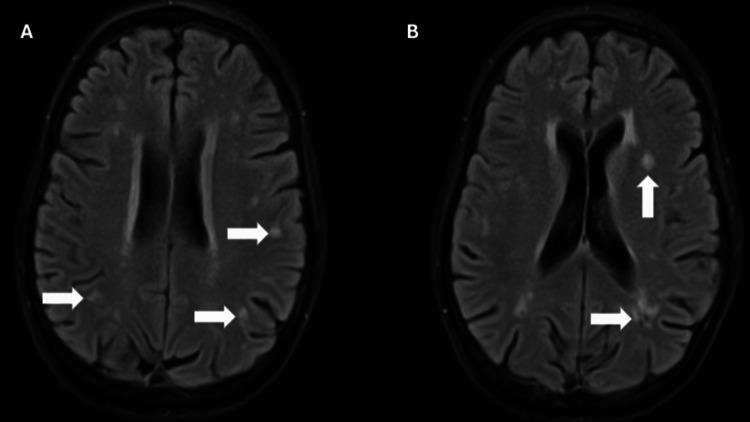
T2 fluid-attenuated inversion recovery (FLAIR) sequence MRI demonstrating multiple subcortical hyperintensities.

The following day, she was noted to have lower extremity weakness, difficulty sustaining a standing posture, and impaired ambulation. Her symptoms further worsened over the next few days with uncontrollable myoclonus, truncal ataxia, and rapid, irregular, conjugate eye movements with horizontal, vertical, and torsional components. Brain magnetic resonance imaging (MRI) with and without contrast revealed nonspecific white matter hyperintensities, as seen in Figure 1. A lumbar puncture was performed, and a cerebrospinal fluid (CSF) paraneoplastic panel was negative, notably for ANNA-1 (Hu), ANNA-2 (Ri), and PCA-1 (Yo). A five-day course of intravenous (IV) methylprednisolone 1 g daily was trialed, as well as a total of 2 g/kg intravenous immunoglobulin (IVIG) split over five days. She was also started on clonazepam 0.5 mg three times daily (TID), valproic acid 1 g TID, and maintenance prednisone 80 mg daily, with little clinical improvement.

After two weeks, the dose of clonazepam was slowly increased to 2 mg TID, and valproic acid was switched to 50 mg of topiramate twice daily, with improvement in eye tracking and resting myoclonus. Her hospital course was complicated by increased emotional lability and agitation, and the decision was made to taper off steroids by 10 mg per day. A follow-up brain MRI revealed no notable abnormalities. A serum paraneoplastic panel eventually resulted negative. She was slowly able to engage in physical and occupational therapy sessions and was discharged to an acute rehabilitation facility. Of note, plasmapheresis and rituximab were discussed but not administered due to the patient’s improvement.

The patient had an interval period where she self-discontinued topiramate and clonazepam, with improvement in cognitive function; however, her opsoclonus and myoclonus quickly returned. She was restarted on clonazepam, which led to improvement in myoclonus with better preserved cognition. Approximately two months later, she underwent a modified radical mastectomy with incomplete dissection of malignant axillary lymph nodes. She was planned to have adjuvant breast and nodal radiation; however, before these interventions could be initiated, she passed away from widely metastatic disease to the lung and liver.

## Discussion

ICIs are immunomodulatory drugs becoming more prevalent in the treatment of metastatic cancers. With the widespread use of these agents, increasing reports of their complications, referred to as IRAEs, are now emerging. Specifically, ICIs carry an increased occurrence of IRAEs that present as neurological disorders and paraneoplastic neurologic syndromes [1,2], making the topic relevant for neurologists and neurology trainees.

ICIs are monoclonal antibodies that function primarily by blocking inhibitory receptors of endogenous immune cells, notably antigen-presenting cells (APCs) and CD4+ T-cells. This, in turn, permits the body’s innate immune cells to mount a response against malignant cells, leading to tumor cell death [3].

**Figure 2:**
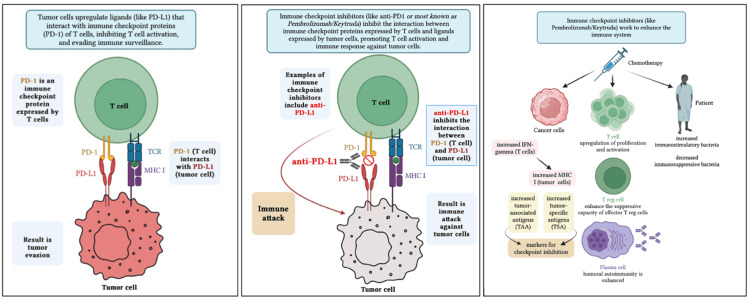
The role of immune checkpoint inhibitors (ICIs) in cancer therapy. Figure credits: The figure was created by Ananta Sriram using Microsoft PowerPoint (Microsoft Corporation, Redmond, WA). PD-1: programmed death-1; PD-L1: programmed cell death ligand 1; TCR: T-cell receptor; MHC I: major histocompatibility complex class I; IFN: interferon.

Two major classes of ICIs exist, each targeting a different immune pathway, as shown below in Figure 2. Cytotoxic T-lymphocyte antigen 4 (CTLA-4) inhibitors, such as ipilimumab, regulate T-cell proliferation in the lymph nodes, an early stage of the immune response. Ipilimumab inhibits CTLA-4 on T-cells from binding to CD80/CD86 on tumor cells, allowing CD28 on T-cells to bind to these receptor molecules instead, potentiating tumor cell death [3]. Conversely, PD-1 inhibitors, such as pembrolizumab and nivolumab, and programmed cell death ligand 1 and 2 (PD-L1/PD-L2) inhibitors work in the later stages of the immune response, within the tumor microenvironment. By blocking either the PD-1 receptor on T cells or PD-L1 on tumor cells, T cell activity is disinhibited [3]. Tumor cells can no longer evade attack, and cancer cell death occurs. PD-1 and PD-L1 inhibitors are used across a broader range of cancers and are generally better tolerated than CTLA-4 inhibitors, which are used primarily in melanomas, and may lead to an increased frequency of IRAEs [4].

IRAEs are thought to be the result of “taking the breaks off” of the immune system, triggering autoimmune reactions within the body [5,6] and potentially activating or exacerbating underlying paraneoplastic syndromes [2,7]. These effects stem from the fact that activated CD4+ T-cells become unregulated, causing an overproduction of endogenous immune cells in both the target tissue and in the periphery. The timing of IRAEs occurs on average two to 12 weeks after initiation of treatment [5].

From a neuroimmunologic perspective, OMAS is thought to involve dysfunction of cerebellar and brainstem circuits, particularly involving Purkinje cells and their inhibitory projections to the deep cerebellar nuclei. Purkinje cells are vulnerable in autoimmune and paraneoplastic syndromes due to their high metabolic activity and expression of intracellular and surface neuronal antigens [8]. In classical paraneoplastic OMAS, both antibody-mediated and cytotoxic T-cell-mediated mechanisms have been implicated [5]. In the setting of immune checkpoint inhibition, enhanced T-cell activation may promote breakdown of peripheral tolerance and expansion of autoreactive CD8+ cytotoxic T-cells targeting neuronal antigens [5]. Additionally, B-cell activation and secondary autoantibody production may occur through epitope spreading or molecular mimicry between tumor-associated antigens and neuronal proteins [5].

OMAS is a well-established paraneoplastic neurological syndrome in breast cancer, and this remains an important consideration in our patient. However, several features raise the possibility of pembrolizumab-related immune dysregulation contributing to symptom onset: (1) temporal association with ICI exposure (within the typical two- to 12-week window for neurological IRAEs) and (2) negative serum and CSF paraneoplastic antibodies. It is important to note that causality cannot be definitively established in this case, and distinguishing between paraneoplastic OMAS and ICI-triggered autoimmunity remains challenging. Nonetheless, this case emphasizes the need for clinicians to remain vigilant for rare neurological complications following ICIs and underscores the importance of early recognition, multidisciplinary management, and careful immunosuppressive treatment planning.

Adult-onset OMAS carries a broad differential diagnosis, including paraneoplastic syndromes, post-infectious autoimmune processes, toxic-metabolic etiologies, and structural brainstem or cerebellar lesions [1]. Structural causes were not supported by neuroimaging in this case, and metabolic and infectious evaluations were unrevealing. Although paraneoplastic OMAS remains a key consideration in breast cancer and may be antibody-negative, the close temporal relationship to pembrolizumab initiation within the expected window of neurological IRAEs raises concern for ICI-associated immune dysregulation [5].

Neurological IRAEs associated with ICIs are rare, affecting approximately 1-6% of patients [6,9]. These adverse events are both central and peripheral, including movement disorders [10], neuro-ophthalmologic conditions [11], and neuromuscular complications such as myasthenia gravis and myositis [9,12]. Other inflammatory diseases reported include encephalitis, myelitis, and aseptic meningitis, all of which can lead to significant morbidity and mortality [13].

Early recognition and prompt treatment are crucial to mitigate the potentially devastating consequences of these neurological adverse effects. Initial management includes discontinuation of the causative agent. Treatment of neurological side effects is controversial, and often requires immunosuppression, which could potentially dampen the antitumor response [4,13,14]. Steroids are often used to manage moderate-to-severe ICI-induced neurotoxicity, with outcomes ranging from full recovery to death if treatment is delayed [14,15]. Other therapies such as IVIG, plasmapheresis, and other monoclonal antibodies like rituximab and infliximab have been used; however, the data for these cases are sparse [13,14,16].

## Conclusions

This case highlights an exceptionally rare and clinically challenging neurological complication occurring in close temporal association with immune checkpoint inhibitor therapy, underscoring a spectrum of immune-mediated neurotoxicity in oncology care. The development of OMAS shortly after the initiation of pembrolizumab, in the absence of identifiable paraneoplastic antibodies, raises considerations regarding immune dysregulation as a direct trigger or an unmasking factor for underlying paraneoplastic processes. As demonstrated, there is an ongoing need for further research to clarify the pathogenesis of IRAEs associated with the breakthrough cancer therapy that ICIs represent. This case emphasizes the need for vigilance when new neurologic symptoms may emerge in patients receiving ICIs, as timely recognition and intervention may influence functional outcomes. Moving forward, it is important for neurologists and trainees to recognize this post-immunotherapy-chemotherapy neurological disease pattern so that we may contribute to the growing literature.
